# Differences in perspectives of pediatricians on advance care planning: a cross-sectional survey

**DOI:** 10.1186/s12904-020-00652-8

**Published:** 2020-09-18

**Authors:** In Gyu Song, Sung Han Kang, Min Sun Kim, Cho Hee Kim, Yi Ji Moon, Jung Lee

**Affiliations:** 1 Department of Pediatrics, Severance Children’s Hospital, Yonsei University College of Medicine, Seoul, South Korea; 2 Department of Pediatrics, University of Ulsan College of Medicine, Asan Medical Center Children’s Hospital, Seoul, South Korea; 3 Department of Pediatrics, Seoul National University Children’s Hospital, 101, Daehak-ro, Jungno-gu, Seoul, 03080 South Korea; 4 College of Nursing, Seoul National University, Seoul, South Korea; 5 Integrative Care Hub, Seoul National University Children’s Hospital, Seoul, South Korea

**Keywords:** Advance care planning, Pediatrician, Palliative care, Prognosis

## Abstract

**Background:**

The increase in the number of pediatric patients with complex health conditions necessitates the application of advance care planning for children. Earlier, withdrawal of life-sustaining treatment was taboo in the medical society in South Korea due to the history of such practice being punishable by law, and physicians tended to pursue aggressive treatment. With changes in public opinion on end-of-life care, the Korean government enacted a new law that protect human dignity by respecting patients’ self-determination and facilitating advance care planning. However, little is known about current state of advance care planning for pediatric patients. The study aimed to assess perceptions regarding advance care planning among South Korean pediatricians and clarify any differences in perception among pediatric subspecialties.

**Methods:**

This study was an observational cross-sectional survey that used a web-based self-report questionnaire. Participants comprised of pediatricians currently caring for children with life-limiting conditions in 2018.

**Results:**

Of the 96 respondents, 89 were included in the analysis. In a hypothetical patient scenario, more hemato-oncologists and intensivists than neonatologists and neurologists preferred to provide comfort care than aggressive treatment. While 72.2% of hemato-oncologists reported that they usually or always discuss advance care plans with parents during treatment, more than half of other pediatricians reported that they seldom do so. Furthermore, 65% of respondents said that they never discuss advance care planning with adolescent patients. Moreover, there were no notable differences among subspecialties. The most prevalent answers to factors impeding advance care planning were lack of systemic support after performing advance care planning (82.0%) and uncertain legal responsibilities (70.8%).

**Conclusions:**

The pediatricians differed in their experiences and attitudes toward advance care planning based on their subspecialty. Consequently, institutional support and education should be provided to physicians so that they can include children and families in discussions on prognosis.

## Background

Advance care planning (ACP) is defined as the process that “enables individuals to define goals and preferences for future medical treatment and care, to discuss these goals and preferences with family and healthcare providers, and to record and review these preferences if appropriate” [1]. ACP can improve the quality of communication between patients and clinicians, increase the application of palliative care, improve patients’ satisfaction and quality of life, and curtail unwanted admissions [2, 3]. Moreover, families have reported that the application of ACP early in the disease process helped them achieve high quality of care [4].

Although various life-limiting pediatric conditions warrant ACP application, such as cancer, extreme prematurity, congenital anomalies, and neuromuscular diseases, several factors cause clinicians to avoid communicating with pediatric patients and families about ACP. These barriers exist for both healthcare professionals and patients’ parents. Lack of knowledge or experience of physicians, uncertain prognoses, and insufficient readiness on the part of parents are known hurdles to be overcome in parent–doctor discussions [5–8]. In particular, prognostic disclosure to pediatric patients has historically been a long-debated issue in ACP. During the 1950s and 1960s, it was recommended to be careful about disclosure; however, since the late 1960s, researchers have been recommending the inclusion of pediatric patients in such discussions. Recently, the recommendation shifted to not considering this decision as a “black-or-white” issue and to balancing conflicting factors on a case-by-case basis [9]. Despite consensus among experts, culture and religion in a society can affect prognostic disclosures to children. The people of South Korea are generally reluctant to disclose disease details to pediatric patients due to their concern that such disclosure may exacerbate illness and impact survival by causing emotional distress [9, 10].

Recently, the Korean government enacted a new law that facilitates ACP. However, to date, only a few studies have examined ACP in pediatric patients [11]. Moreover, depending on the characteristics of patients and diseases, few studies have been conducted on the difference of opinions on ACP among the various subspecialties of pediatrics. Hence, this study aimed to assess pediatricians’ perceptions regarding ACP and barriers to the implementation of ACP in pediatric patients. We hypothesize that perceptions regarding ACP differ among pediatric subspecialties and attempt to verify this hypothesis.

## Methods

### Study design and study population

An observational cross-sectional online survey was conducted to assess South Korean pediatricians’ perceptions of ACP. A web-based self-report questionnaire was administered to pediatricians of neonatology, neurology, critical care medicine, and hemato-oncology subspecialties, since they all often treat patients with life-limiting conditions.

### Questionnaire design and development

The survey instrument for pediatric ACP was evaluated by reviewing relevant reports, papers, and statutes [5–8]. Questions were reviewed by pediatric doctors, nurses and social workers who usually care for children with life-threatening diseases. ACP was defined in the beginning of the questionnaire since pediatricians in Korea in general are not familiar with the concept of ACP. The instrument comprised five major domains: 1) Demographic questions, background subspecialty, and career as a pediatrician (duration after obtaining certificate of pediatrics); 2) preference in decision-making and timing of discussions on providing life-sustaining treatment in two scenarios; 3) six items pertaining to pediatricians’ experiences in making decisions regarding life-sustaining treatment; 4) two items related to the barriers to ACP implementation in children and adolescents and its weights; and 5) three items on attitudes toward legal issues (Act on Hospice and Palliative Care and Decisions on Life-Sustaining Treatment for Patients at the End-of-life). The survey was pilot tested with two pediatricians and revised according to their feedback. Finally, participants took approximately 10 min to complete the online survey.

### Data collection

Using the web-based survey (SurveyMonkey.com, Palo Alto, CA), data were collected during October and November 2018. Invitations to participate in the survey signed by the president of the Seoul National University Hospital and the investigators were e-mailed to the Korean Society of Pediatric Hematology-Oncology, Korean Society of Neonatology, Korean Society of Pediatric Critical Care Medicine, and Korean Child Neurology Society. Subsequently, each society distributed the invitation letters and the survey’s web address to potential participants. The respondents’ confirmation to participate was considered informed consent.

Each society sent reminder e-mails to nonrespondents through pediatric societies 2 weeks after sending the invitations. The pediatricians who declined the invitations were not subsequently contacted and respondents’ information, such as e-mail addresses, names, and place of employment, were not linked to their replies.

### Measures

The primary outcome, including the participating pediatricians’ perceptions of ACP, was obtained from the survey by four out of the five major domains of the survey instrument. They included experience in pediatric ACP, legal attitude, decision-making ability, and timing preferences regarding clinical scenarios. The study considered two scenarios and both considered six-year-old male patients requiring intubation (Supplementary file 1). The first scenario involved a hypoxic-ischemic encephalopathy patient that represented chronic conditions. The second scenario referred to a leukemia patient who represented intractable cancer patients. Both scenarios were created and reviewed by a multidisciplinary team who cared for children with life-limiting conditions. In survey question 1, respondents were asked to select one of the two choices for the aforementioned two cases. In both the cases, answer 1 was offering invasive medical treatment despite the decreased possibility of survival and answer 2 referred to providing comfort care, rather than aggressive but less effective treatment. To verify the secondary outcome, that is, revealing the differences in perception among specialties, the collected data were classified and analyzed according to each participant’s specialty and career as a pediatrician. Additionally, the survey clarified participants’ perceptions of barriers to ACP in pediatric patients.

### Statistical analysis

Statistical tests were conducted using SPSS version 21.0 (SPSS Inc./IBM, Chicago, IL) and STATA version 15.1 (StataCorp, College Station, TX). Respondents’ replies, as well as demographics, were described using means and frequencies. The demographic data were organized into categorical data for analysis. To measure outcomes, we compared means and frequencies among groups and adjusted proportion codes were used (adjusted for age, sex, religion, and career as a pediatrician, as well as pediatric ACP education).

## Results

### Participant characteristics

A total of 966 e-mail invitations were distributed through each of the four pediatric societies. Among all eligible pediatricians, 96 responded to the survey (response rate: 9.9%). Of these, the study excluded five with incomplete demographic data and two who did not obtain the survey via the society. The data of the remaining 89 respondents were included in the analysis. Table 1 depicts the respondents’ characteristics. The table clarifies that the majority of the respondents had neonatology as their specialty, followed by hemato-oncology, neurology, and intensive care medicine in decreasing order. There were no differences in the distributions of sex, age, religion, or career as a pediatrician among respondents’ specialties. However, more pediatric hemato-oncologists were educated on pediatric ACP than the other specialties (Table 1).

**Table 1:** Demographics of survey participants (*n* = 89)

	Neurology n (%) (*n* = 10)	Neonatology n (%) (*n* = 54)	Intensive Care n (%) (*n* = 7)	Hemato-Oncology n (%) (*n* = 18)
Sex				
Male	6 (60.0)	14 (25.9)	2 (28.6)	5 (27.8)
Female	4 (40.0)	40 (74.1)	5 (71.4)	13 (72.2)
Age (years)				
30–39	5 (50.0)	18 (33.3)	5 (71.4)	10 (55.6)
40–49	3 (30.0)	26 (48.1)	2 (28.6)	4 (22.2)
50–59	1 (10.0)	5 (9.3)	0 (0)	4 (22.2)
≥ 60	1 (10.0)	5 (9.3)	0 (0)	0 (0)
Religion				
Protestant	6 (60.0)	20 (37)	3 (42.9)	7 (38.9)
Catholic	1 (10.0)	12 (22.2)	1 (14.3)	5 (27.8)
Buddhist	1 (10.0)	5 (9.3)	1 (14.3)	1 (5.6)
None	2 (20.0)	17 (31.5)	2 (28.6)	5 (27.8)
Career as a pediatrician				
≤ 10 years	7 (70.0)	32 (59.3)	5 (71.4)	12 (66.7)
> 10 years	3 (30.0)	22 (40.7)	2 (28.6)	6 (33.3)
Had education about pediatric advance care planning				
Yes	0 (0)	8 (14.8)	2 (28.6)	10 (55.6)
No	10 (100)	46 (85.2)	5 (71.4)	8 (44.4)

Notes: n refers to the number of respondents in each category

### Preference in decision-making for two life-sustaining treatment scenarios

There were differences among subspecialties in the decisions made to provide life-sustaining treatment. Pediatric hemato-oncologists were highly inclined to provide comfort care (answer 2) in both scenarios. The adjusted proportions for answer 2 were 83.0% for case 1 and 93.9% for case 2 among pediatric hemato-oncologists. Pediatric intensivists preferred answer 2 in both cases as well. Among them, approximately 57% chose comfort care for case 1 and all seven respondents chose answer 2 for case 2. Contrarily, a lesser number of neonatologists and neurologists chose comfort care for both cases (Table 2).

**Table 2:** Results of decision-making on the two presented cases (preference for comfort care)

Subspecialties	n	Proportion (%)	Adjusted Proportion (%)
**Case 1 (HIE)**			
Neurology (*n =* 10)	2	20.0	10.3
Neonatology (*n =* 54)	27	50.0	52.0
Intensive care (*n =* 7)	4	57.1	57.1
Hemato-oncology (*n =* 18)	14	77.8	83.0
**Case 2 (Leukemia)**			
Neurology (*n =* 10)	3	30.0	35.7
Neonatology (*n =* 54)	26	48.1	48.1
Intensive care (*n =* 7)	7	100	100
Hemato-oncology (*n =* 18)	17	94.4	93.9

*HIE* hypoxic-ischemic encephalopathy.

Notes: The proportions were adjusted for age, sex, religion, and career as a pediatrician, as well as pediatric advance care planning education. Answer 1, preference for invasive respiratory support, including intubation and mechanical ventilation in the specified cases; Answer 2, preference for antibiotics and medications for symptom control rather than invasive respiratory support, in the cases

### Preference of time point to discuss advance care planning

**Fig. 1:**
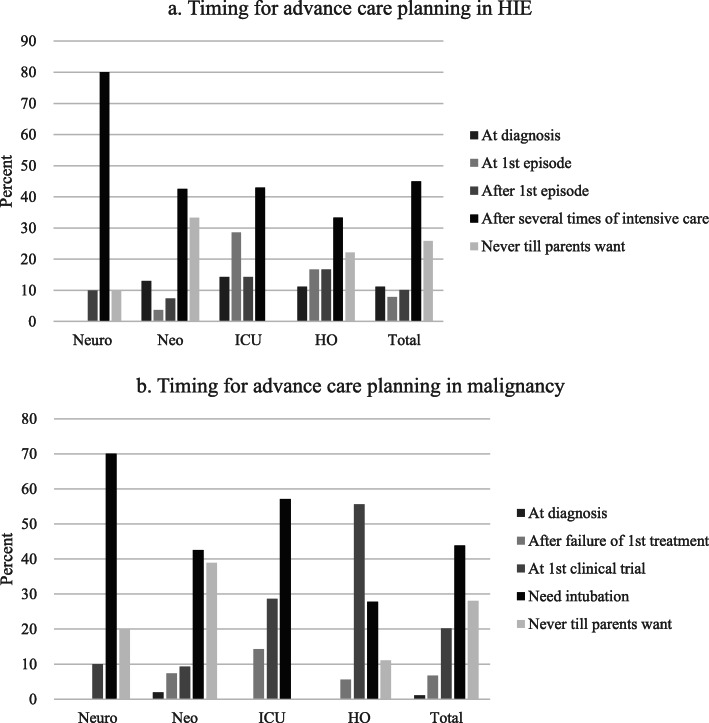
Preferred timing to implement advance care planning: HIE, hypoxic ischemic encephalopathy; Neuro, Neurology; Neo, Neonatology; ICU, intensive care unit; HO, hemato-oncology

Respondents were asked to specify the time point preferred by them to discuss ACP. For both treatment scenarios, the majority of the pediatricians preferred to discuss ACP after the patient had already experienced multiple events of invasive ventilator care or when the patient needed intubation (case 1—40/89, 44.9%; case 2—39/89, 43.8%). However, the proportion of respondents who preferred to discuss ACP in an earlier stage (first three time points) of disease was higher among hemato-oncologists and intensivists than among neonatologists or neurologists (Supplementary Table 1). In both cases, more than 30% of neonatologists reported that they would not initiate any discussion on ACP until parents displayed their willingness to discuss it (Fig. 1).

### Discussion on advance care planning with patients’ parents in advance

Respondents were asked how often they discussed ACP with patients’ parents in case their patients had a high possibility of death within a few years. Their answers differed among subspecialties. While 90% of pediatric neurologists and more than 50% of pediatric intensivists and neonatologists answered that they rarely or never discussed ACP with parents, more than 70% of pediatric hemato-oncologists stated that they discussed ACP with parents mostly or always. Comparing the results on the basis of career as a pediatrician, those with career durations of 10 years or less discussed ACP more frequently with parents than those with longer career durations (Table 3, Supplementary Tables 2 and 3).

**Table 3:** Results of discussion on advance care planning ahead with parents

	None	Rarely	Mostly	Always
Specialty				
Neurology (*n =* 10)	1 (10.0%)	8 (80.0%)	1 (10.0%)	0 (0%)
Neonatology (*n =* 54)	8 (14.8%)	23 (42.6%)	20 (37%)	3 (5.6%)
Intensive care (*n =* 7)	1 (14.3%)	3 (42.9%)	1 (14.3%)	2 (28.6%)
Hemato-oncology (*n =* 18)	0 (0%)	5 (27.8%)	9 (50%)	4 (22.2%)
Career as a pediatrician (years)				
≤ 10 (*n* = 56)	1 (1.8%)	29 (51.8%)	19 (33.9%)	7 (12.5%)
> 10 (*n* = 33)	9 (27.3%)	10 (30.3%)	12 (36.4%)	2 (6.1%)
Had education about pACP				
Yes (*n* = 20)	1 (5.0%)	5 (25.0%)	9 (45.0%)	5 (25.0%)
No (*n* = 69)	9 (13.0%)	34 (49.3%)	22 (31.9%)	4 (5.8%)
Total (*N =* 89)	10 (11.2%)	39 (43.8%)	31 (34.8%)	9 (10.1%)

*pACP* pediatric advance care planning

### Discussion on advance care planning ahead with adolescent patients

Respondents were asked whether they discussed ACP ahead with adolescent patients. More than 60% of pediatricians “never” discussed ACP with adolescent patients. Furthermore, there was no notable difference among specialties, career, and education (Table 4).

**Table 4:** Results of discussion on advance care planning with adolescent patients

	None	Rarely	Mostly	Always
Subspecialty				
Neurology (*n* = 9)	8 (88.9%)	0 (0%)	1 (11.1%)	0 (0%)
Intensive care (*n* = 7)	5 (71.4%)	2 (28.6%)	0 (0%)	0 (0%)
Hemato-oncology (*n* = 18)	9 (50.0%)	8 (44.4%)	1 (5.6%)	0 (0%)
Career as a pediatrician (years)				
≤ 10 (*n* = 23)	16 (69.6%)	6 (26.1%)	1 (4.3%)	0 (0%)
> 10 (*n* = 11)	6 (54.6%)	4 (36.4%)	1 (9.1%)	0 (0%)
Had education about pACP				
Yes (*n* = 12)	6 (50.0%)	5 (41.7%)	1 (8.3%)	0 (0%)
No (*n* = 22)	16 (72.7%)	5 (22.7%)	1 (4.6%)	0 (0%)
Total (*N* = 34)	22 (64.7%)	10 (29.4%)	2 (5.8%)	0 (0%)

*pACP* pediatric advance care planning

### Perception of barriers to advance care planning

**Fig. 2:**
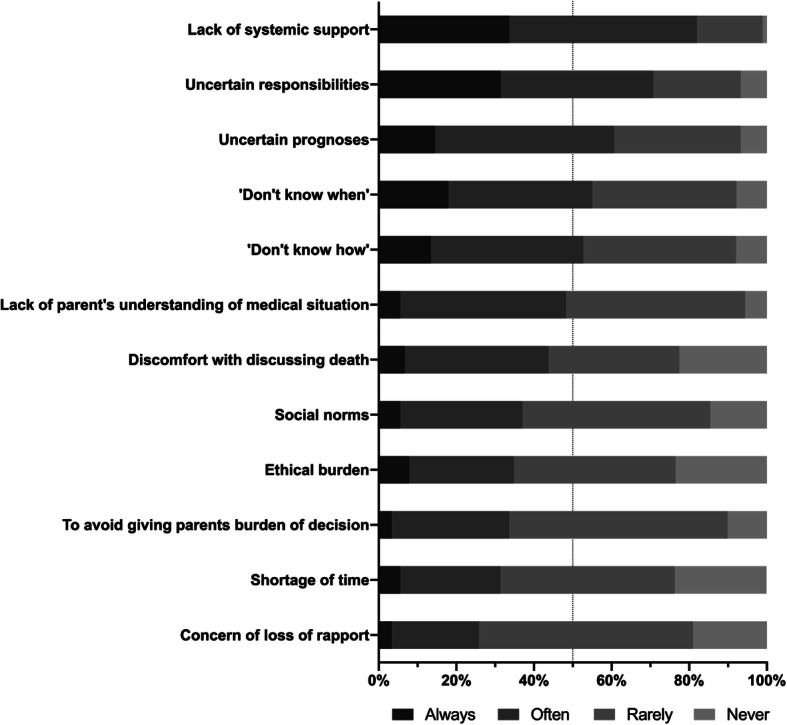
Barriers to advance care planning

Barriers to ACP were rated according to respondents’ identification as often or always a barrier. The three highest-rated barriers were “lack of systemic support after ACP (palliative care or family support program)” (82.0%), “uncertain responsibilities” (70.8%), and “uncertain prognosis” (60.7%). Furthermore, more than 50% of pediatricians identified “do not know-when” (55.1%) or “do not know-how” (52.8%) as occurring often or always. Conversely, more than 50% of respondents specified six issues that rarely or never acted as barriers to ACP: “concern of loss of rapport” (74.2%), “shortage of time” (68.5%), “to avoid giving parents burden of decision” (66.3%), “ethical burden” (65.2%), “social norms” (62.9%), and “discomfort with discussing death” (56.2%; Fig. 2).

## Discussion

### Main findings

To the best of our knowledge, this study conducted the first survey to evaluate pediatricians’ perspectives and opinions on ACP in South Korea. In addition, only a few earlier studies have analyzed differences in perceptions according to the pediatricians’ subspecialty. In this study, we found that preferred treatments for respiratory difficulties or timing for providing ACP of patients with life-limiting conditions were different for different subspecialties. Furthermore, although pediatric hemato-oncologists discussed ACP more than other pediatricians, only a few, regardless of subspecialty, had experience in applying ACP among adolescent patients. Finally, the lack of systemic support after providing ACP was the prevalent barrier to ACP implementation.

A previous study revealed that over 90% of parents stated that palliative care was appropriate for children who were less likely completely recover. We reflected this in the scenarios in which parents were open to comfort or palliative care [12]. We also assumed that there would be a difference in pediatricians’ choices between disease groups because hospice and palliative care policy in South Korea focused on cancer patients alone until 2017 [13]. In both hypothetical scenarios, pediatric hemato-oncologists chose comfort care more often and preferred earlier timing for ACP more than other pediatricians (Table 2; Fig. 1). Furthermore, they held ACP discussions with parents more often than other specialties. These results may be explained by the difference in the proportions of doctors who had received prior education on ACP (Table 1). This assumption is supported by the result that difference in timing was reduced after controlling for education experience. Due to the fact that the national policy for palliative care has not been implemented equally across all subspecialties, hemato-oncologists might have had relatively more opportunities to have received ACP education [13]. Case 2 (the leukemia patient) revealed the differences in perceptions among specialties more distinctly and indicated that both pediatric intensivists and hemato-oncologists preferred comfort care more often than neurologists or neonatologists. Disease trajectory of cancer is more predictable than non-cancer diseases and these two subgroups of pediatricians who usually care for refractory leukemia patients may have more experience for making decisions that do not involve invasive respiratory support [14]. According to the Act on Hospice and Palliative Care and Decisions on Life-Sustaining Treatment for Patients at the End of Life, life-sustaining treatment can be withdrawn or withheld only for patients in dying process [11]. In the survey, although questions encouraged them to select answers without considering the law, neurologists and neonatologists who were not familiar with refractory leukemia patients chose more conservative answers based on the applicable law.

In this study, 55.0% of the respondents answered that they never (11.2%) or rarely (43.8%) held ACP discussions with parents; the proportion was comparable to, or even higher, than in previous studies [4, 15–17]. Earlier, withdrawal of life-sustaining treatment was taboo within the medical society in South Korea due to the practice historically being punishable by law, and physicians tended to pursue aggressive treatment. Accordingly, ACP was rarely performed and treatment decisions often did not reflect patients’ or their families’ value. However, changes in public opinion on end-of-life care resulted in the enactment of a new law to create an environment that respects patients’ autonomy [11, 18]. This social change affected medical practice and younger doctors became more open to making treatment plans with patients’ parents as report the following results: 27.3% of pediatricians whose career duration was more than 10 years, but only 1.8% of junior pediatricians, did not conduct ACP discussions with parents (Table 3; Supplementary Table 3).

Despite the changing trends, most pediatricians seem to find it extremely difficult to discuss prognosis or life sustaining treatment options directly with patients which is consistent with previous studies [19–21]. In the current study, over 90% of the respondents replied that they had never (64.7%) or rarely (29.4%) discussed ACP with their adolescent patients. It is known that engagement of children and adolescents can benefit their ACP and the new law provisions indicate that any patient can request ACP discussion regardless of their age, and doctors should respond to that. Nevertheless, there is no guidance on how to communicate with pediatric patients regarding their end-of-life care planning [11, 19–21]. It is questionable whether just enacting the law can make a difference and future studies are needed to assess the performance change in adolescent ACP.

Survey responses indicated that the absence of an effective support system, such as palliative care or family support teams, is a major barrier to ACP, followed by uncertain legal responsibility, uncertain prognosis, and lack of knowledge about ACP. An earlier study in the United States reported that parental factors (unrealistic expectation, insufficient understanding of prognosis, and lack of readiness) are the most prevalent barriers to ACP [8]. Further, Korean oncologists (internal medicine) also opined that familial factors (reluctance, hope, and conflict) and unclear prognosis are more frequent barriers than a lack of systemic support or scarcity of knowledge, which correspond to the results of the current study [22]. According to the result, only 20 participants (22.4%) received appropriate education in this matter (Table 1) and 55.0% were lacking in confidence regarding pediatric ACP (data not shown). Furthermore, until 2018, only a few hospitals had provided palliative care for pediatric patients; therefore, pediatricians may be concerned about how to have a discussion and management after ACP, and believe they can do nothing more to support their patients and families. The establishment of a consultation or palliative team is known to facilitate ACP discussion and provide comfort to healthcare professionals [23]. In 2018, the South Korean government initiated a national pilot program to fund the establishment of pediatric palliative care teams in hospitals. The program is expected to be extended to hospitals that mainly treat children requiring pediatric palliative care, and further research is required on whether the extension of the palliative care program will help lower the barriers to ACP [13].

The implementation of an education program for pediatricians will also be helpful in overcoming these barriers. Medical staffs should have competence and knowledge on how to begin and facilitate communication related to care planning and when to consult the pediatric palliative care team. To do that, implementing an education program during residency training is necessary. Bagatell, Meyer, Herron et al. indicated that pediatric residents who had received appropriate education were significantly more comfortable handling death-related logistic issues and more familiar with symptom management and communicating about death or end-of-life care with colleagues and families than residents with less education [24]. In addition, ACP tools or guidance mechanisms are known to enhance communication between young people and healthcare providers in the ACP process [20, 21]. Accordingly, we developed the South Korean version of ACP tools and a practical guide for pediatric ACP by referring to preexisting tools and expect these measures to facilitate effective communication [25].

### Strengths and limitations

This was the first study on pediatricians’ perceptions of and attitudes toward ACP in South Korea. In particular, since this survey was conducted at the initial stages of enacting the new law on end-of-life care and the national pediatric palliative pilot program, we expect that this study can provide baseline data to evaluate the effectiveness of new policies. Furthermore, we analyzed the data based on pediatric subspecialties and found different opinions on ACP, which implies the necessity of establishing a priority for ACP education.

However, the study had some limitations. First, since the invitation to participate in the survey was sent to all members of four South Korean pediatric societies, the study’s response rate was low and caution is needed in interpreting the results as an observational study. Nevertheless, the present method of participation was one of the few options accessible to pediatricians who treat patients with life-limiting conditions in situations where personal information cannot be obtained. Second, we were not able to enroll pediatricians in some subspecialties, such as cardiology and nephrology, in the study due to the low interest of the society. By analyzing their opinions, we could have evaluated more diverse perceptions of ACP since they frequently deal with chronic diseases. In order to address these limitations, further studies should include more subspecialties with representative sample size. Furthermore, qualitative studies that assess factors which support or serve as barriers for ACP is added would provide more conclusive results.

## Conclusions

This study revealed that pediatricians’ experiences and opinions regarding ACP could be different according to their subspecialty and the absence of a support system acts as a barrier to ACP conversation. These results suggest that it is necessary to provide systemic support and educate pediatricians about ACP to facilitate the establishment of a treatment goal in the patient’s best interests. Furthermore, more investigations must be conducted on the impact of the newly enacted law and palliative program on pediatricians’ perceptions and behaviors.

## Supplementary information

**Additional file 1.**

**Additional file 2.**

## Data Availability

The datasets used and/or analyzed during the current study are available from the corresponding author on reasonable request.

## Abbreviations

ACP: Advance care planning
HIE: Hypoxic ischemic encephalopathy
Neuro: Neurology
Neo: Neonatology
ICU: Intensive care unit
HO: Hemato-oncology
