# Supplementary material for: Differences in perspectives of pediatricians on advance care planning: a cross-sectional survey
Source: BMC Palliat Care. 2020 Sep 18;19:145. doi: 10.1186/s12904-020-00652-8 (PMC7500719; doi:10.1186/s12904-020-00652-8)
Supplement: Supplementary file 1 — Additional file 1. [file 12904_2020_652_MOESM1_ESM.docx]

Hypothetical patient scenarios

Case 1.

A 6-year old boy was diagnosed with hypoxic-ischemic encephalopathy with dystocia at 40 weeks gestational age. Although the boy was able to spontaneously open his eyes, eye contact and communication were impossible. The boy is currently on medication to control severe rigidity. When being cared for at home, the boy requires airway suctions two to three times a day with continuous oxygen supply through a nasal prong of 1 L/min. He has been treated for multiple events of aspiration pneumonia over the last two years. Mechanical ventilation, along with tracheostomy, was applied during the treatment. Since his last time in intensive care, the boy’s parents did not want him to undergo any pain, and stated that they did not want intubation or mechanical ventilation care in case of future respiratory difficulty.

This boy was admitted to the emergency center with a complaint of respiratory difficulty and fever. Despite 10 L/min of oxygen, the boy’s oxygen saturation was only 82 %, serum carbon dioxide was as high as 95 mmol/L, and the chest x-ray revealed pneumonia. The boy’s parents refused intubation, and wanted him to receive antibiotics and medications for pain management only.

Case 2.

A 6-year old boy with leukemia received conventional chemotherapy but a complete remission was not achieved. As additional multi-drug chemotherapy was also ineffective, the medical staff attempted to control the boy’s leukemia through a therapeutic first-phase clinical trial. However, due to viral hepatitis, the chemotherapy had to be stopped.

The boy developed complicated pneumonia with massive pleural effusion, which required simultaneous intubation and mechanical ventilation. However, the boy’s parents did not want any further treatment, and only wanted for their son to be more comfortable and without pain. The medical staff of the pediatric hemato-oncology department has assumed that the boy’s disease is unlikely to be cured.
