# Supplementary material for: Differences in perspectives of pediatricians on advance care planning: a cross-sectional survey
Source: BMC Palliat Care. 2020 Sep 18;19:145. doi: 10.1186/s12904-020-00652-8 (PMC7500719; doi:10.1186/s12904-020-00652-8)
Supplement: Supplementary file 2 — Additional file 2. [file 12904_2020_652_MOESM2_ESM.docx]

Supplementary Table 1. Preferred earlier timing to implement advance care planning (after adjustment)

|  | | Proportion (%) | Adjusted proportion (%) |
| --- | --- | --- | --- |
| Scenario 1 (HIE) |  |  |  |
| Neurology or neonatology (n = 64) | | 21.9 | 22.5 |
| Intensive care or  hemato-oncology (n = 25) | | 48.0 | 40.4 |
| Scenario 2 (malignancy) | |  |  |
| Neurology or neonatology (n = 64) | | 62.5 | 68.3 |
| Intensive care or  hemato-oncology (n = 25) | | 92.0 | 89.4 |

Notes: The proportions were adjusted for age, sex, religion, career as a specialist, and pediatric ACP education. HIE, hypoxic ischemic encephalopathy.

Supplementary Table 2. Results on frequency of discussing advance care planning ahead with parents by subspecialty (after adjustment)

| Subspecialty | None or Rarely (%) | Mostly or Always (%) |
| --- | --- | --- |
| Neurology (n = 10) | 89.9 | 10.1 |
| Neonatology (n = 54) | 56.5 | 43.5 |
| Intensive care (n = 7) | 55.5 | 44.5 |
| Hemato-oncology (n = 18) | 31.0 | 69.0 |

Note: The proportions were adjusted for age, sex, religion, and career as a specialist, as well as pediatric ACP education.

Supplementary Table 3. Results on frequency of discussing advance care planning ahead with parents by career as a pediatrician (after adjustment)

| Career as a pediatrician (years) | None (%) | Rarely, Mostly, or Always (%) |
| --- | --- | --- |
| ≤10 (n = 56) | 1.2 | 98.8 |
| >10 (n = 33) | 23.4 | 76.6 |

Note: The proportions were adjusted for sex, religion, subspecialty, and pediatric ACP education.
